# PROs Assessment in Dual (Either Oral or Injectable) Antiretroviral Regimen in People with HIV: A Narrative Review

**DOI:** 10.3390/v18010007

**Published:** 2025-12-19

**Authors:** Maria Mazzitelli, Olivia Bargiacchi, Maria Aurora Carleo, Andrea Giacomelli, Camilla Muccini, Lucia Taramasso, Marcello Trizzino, Antonella Cingolani

**Affiliations:** 1 Dipartimento di Sicurezza e Bioetica, Sezione di Malattie Infettive, Università Cattolica del Sacro Cuore, 00168 Rome, Italy; 2 Dipartimento di Scienze Mediche e Chirurgiche, Fondazione Policlinico Universitario Agostino Gemelli IRCCS, 00168 Rome, Italy; 3 Infectious and Tropical Diseases Unit, Ospedale Maggiore della Carità, 28100 Novara, Italy; 4 Infectious Diseases and Gender Medicine Unit, Cotugno Hospital, 80131 Napoli, Italy; 5 Department of Biomedical Sciences, Milan University, 20122 Milan, Italy; 6 Infectious Diseases Unit, ASST Fatebenefratelli Sacco, 20157 Milan, Italy; 7 Infectious Diseases Unit, Ospedale San Raffaele IRCSS, 20132 Milano, Italy; 8 Infectious and Tropical Diseases Unit, Ospedale San Martino, 16131 Genova, Italy; 9 Infectious and Tropical Diseases Unit, Azienda Ospedaliero-Universitaria Policlinico Giaccone, 90127 Palermo, Italy; marcello.trizzino@policlinico.pa.it

**Keywords:** PROs, patient-reported outcomes, HIV, people with HIV, two-drug regimens, injectables

## Abstract

Background: With modern antiretroviral regimens, durable viral suppression is now achieved in most people with HIV (PWH), whose life expectancy approaches that of the general population. Consequently, recent guidelines emphasise, beyond virological and immunological control, health-related quality of life and patient-reported outcomes (PROs) as targets of HIV care, including for dual regimens. Methods: We conducted a narrative review of clinical trials and observational studies evaluating PROs in adults treated with dual antiretroviral therapies, either oral or long-acting injectable. We also examined guideline documents and implementation studies addressing the role, feasibility, and interpretation of PROs in routine HIV care. Results: Trials of dual regimens reported high treatment satisfaction, convenience, and stable or improved quality of life, with some concerns related to injection-site reactions and visit burden for long-acting formulations. Emerging real-world data broadly confirm these findings but remain heterogeneous, with variability in instruments, assessment timing, and analytic approaches, limiting comparability and clinical use. Conclusions: PROs may support shared decision-making and optimise the use of dual therapies in PWH, but their uptake in clinical practice is still limited. Standardised tools, clearer interpretative frameworks, and pragmatic implementation strategies are needed to better integrate PROs into everyday HIV care.

## 1. Introduction

Thanks to a great innovation in the field of antiretroviral therapy, viral suppression achievement is no longer an issue in people with HIV (PWH), who are able to reach a life expectancy overlapping that of the general population [1]. As a consequence, different needs and clinical challenges have emerged over time, such as the management of chronic comorbidities, copying within polypharmacy and drug interactions, and dealing with the aging process and its complications. Moreover, many years have passed since UNAIDS stated that at least 90% of PWH who achieved virological suppression should reach a good quality of life (QoL) in 90% cases [2]. QoL is very difficult to assess in routine clinical practice, requiring dedicated time and efforts quite often not available due to resource limitations, understaffing, and time constraints [3]. Notwithstanding, patient-reported outcomes (PROs) and their measures (PROMs) in routine outpatient practice is emerging as a major clinical outcome [4]. Even the recent EACS guidelines for the first time introduced a yearly PRO assessment both for symptom burden and QoL in PWH [5]. PROs are something that people report regarding their own health, wellbeing, and QoL associated with healthcare systems and treatment which is not influenced by the care provider’s interpretations [6]. PROs encompass a broad range of parameters that reflect the patient’s own perception of their health, including symptoms, treatment side effects, physical and cognitive functioning, emotional wellbeing, social participation, and overall health-related quality of life. PROs also frequently capture treatment satisfaction, adherence challenges, and the daily impact of chronic conditions. In research and clinical settings, PRO assessment is usually conducted using validated questionnaires or scales—administered electronically, on paper, or through interviews—that allow patients to directly report their experiences without external interpretation. These instruments are applied at baseline and during follow-up visits to monitor changes over time, evaluate treatment effects, and support patient-centred decision-making. Importantly, new clinical trials and observational studies are adding these parameters to HIV viral load and blood test results when assessing the efficacy and tolerability of a new treatment. Moreover, increasing attention to PROs and PROMs is emerging from patient, clinical, and pharmaceutical perspectives, since including the patient’s perspective as the outcome of both observational research and randomised clinical trials allows us to gain more insights and a holistic vision on the overall benefit of any interventions [7]. The relevance of PRO assessment becomes particularly evident when comparing three-drug regimens (3DRs) with newer two-drug regimens (2DRs) in PWH. Traditional 3DRs, while highly effective, may carry a greater pill burden (despite this issue being minor today since many triple therapies are also available as single-tablet regimens), higher risk of cumulative toxicities, and more potential for drug–drug interactions, all of which can negatively influence daily functioning and overall QoL. In contrast, 2DRs were developed to maintain virologic efficacy while reducing medication exposure, simplifying treatment, and potentially improving tolerability and long-term safety. These anticipated advantages created a strong rationale for incorporating PRO assessment into 2DR clinical trials, as understanding patients’ subjective experiences is essential to determine whether simplified regimens translate into meaningful improvements in wellbeing.

Hence, assessing PROs in PWH has become increasingly important, as these measures can capture dimensions of wellbeing that extend beyond virologic suppression and mostly involve people’s wellbeing and perceptions. Different antiretroviral therapy regimens can have distinct effects on symptoms, treatment satisfaction, mental health, and daily functioning, thereby differentially shaping the overall quality of life of PWH. Incorporating PRO assessments therefore provides a more comprehensive understanding of treatment impact and supports patient-centred decision-making in clinical practice. However, several issues remain about their implementation in real-life clinical settings (time constraints during routine visits, participant’s survey fatigue, reduced motivation, limited literacy, etc.), and about which are the most reliable and useful tools to be used. Moreover, the introduction of newer and innovative antiretroviral treatment, such as 2DRs, either injectable or oral, revolutionised the traditional therapeutic approach. The objective of this narrative review is to summarise the evidence on the use of PROs in PWH who are simplifying their 3DR to a 2DR, either oral or long-acting, assessing differences in PROs either in RCTs and observational studies.

## 2. Materials and Methods

The research followed an evidence-based consensus methodology, which involved nominal group meetings and a narrative literature review. The study was carried out in accordance with the Declaration of Helsinki and the principles of Good Clinical Practice. Due to the non-sensitive nature of the data collected, ethical committee approval was not required. We searched the main database (PubMed, Embase, and Scopus) with the keyword “PROs” or “patient-reported outcomes” and “HIV” or “PWH” or “PWH” and “dual therapy” or “two-drug regimen” or “long-acting” or “cabotegravir/rilpivirine” (CAB/RPV) or “dolutegravir/lamivudine” (DTG/3TC) or “dolutegravir/rilpivirine” (DTG/RPV). We included all randomised clinical trials and observational studies ever published from the publication of the first dual strategy as a switch (March 2018) to April 2024. Four reviewers independently screened the abstracts to determine eligibility for full-text review (A.G., L.T., C.M., O.B.). Neither geographical nor language restrictions were applied. Studies were included if they met all the following criteria: (i) study published in full; (ii) study describes the use of PROs; (iii) study includes people with HIV who received as a switch strategy either an oral (DTG/RPV or DTG/3TC) or injectable (CAB/RPV) dual regimen. Exclusion criteria, applied on the results retrieved, were as follows: (i) papers in the form of abstract, conference report, or poster; (ii) abstract not available; (iii) full-text papers not accessible. In case no published data were available, we considered conference abstract. A table was elaborated with all the PROs under assessment in RCTs investigating CAB/RPV treatment in PWH, with the following fields: study phase, intervention and comparison, number of participants, type of end point, type of PRO instrument, and outcome. We lastly assessed, described, and discussed factors related to an improvement in PROs.

## 3. Results

### 3.1. Treatment Satisfaction in Clinical Trials of Oral Two-Drug Regimens

Oral 2DR simplified multi-drug HIV treatment by reducing pill burden, streamlining drug regimens, and improving adherence [8,9], a key precursor of which is treatment satisfaction. In clinical trials, PROs related to treatment satisfaction are commonly assessed through validated questionnaires, such as the HIV Treatment Satisfaction Questionnaire (HIVTSQ), that includes questions covering treatment convenience, ease of use, side effects, and overall satisfaction; the 10-item HIVTSQ, with scores ranging from 0 to 60, is typically administered as a self-reported questionnaire, allowing patients to provide direct feedback on their treatment experience [10]. SALSA was a randomised (1:1) trial conducted on virologically suppressed PWH on a 3-/4-drug (3/4DR) regimen who switched to DTG/3TC or continued their current antiretroviral regimen (CAR); overall, 39% were women, the median age was 45 years, and the median CD4 cell count was 669 cells/mm3 [11]. Treatment satisfaction was evaluated by age: From baseline, between-group differences in the HIVTSQs total score were significantly higher in participants aged ≥50 years in the DTG/3TC group compared to those in the 3/4DR group at weeks 4 (1.6, 95%CI 0.3–3.0) and 24 (1.3, 95%CI 0.1–2.5) [12]. Participants aged <50 years who switched to DTG/3TC increased their HIVTSQs total score more than people in the same age group who continued their CAR at all time points assessed (week 4 between-group difference: 1.8, 95%CI 0.7–2.9; week 24: 1.5, 95%CI 0.6–2.4; week 48: 1.7, 95%CI 0.7–2.7) [12]. Looking at the percentage change from baseline in the proportion of participants with the highest score of 6/6 on individual HIVTSQ items up to week 48, the greatest percentage increases throughout the study for people in the DTG/3TC vs. 3/4DR group were in the areas of treatment recommendation (21% vs. 3%), side effects (25% vs. 7%), lifestyle (25% vs. 7%), and willingness to continue treatment (47% vs. 17%) [13].

In line with the previous findings, significant improvements in treatment satisfaction had also been observed in the SWORD-1 and -2 trials, two identically designed, randomised (1:1) studies assessing the efficacy and safety of switching to DTG/RPV compared with continuing CAR in PWH under virological suppression [14]. Although the mean (standard deviation, SD) treatment satisfaction scores were high at baseline in both the DTG/RPV and CAR groups [54.4 and 53.9, respectively], modest but statistically significant changes were described in the ART switch arm compared with the control arm in the HIVTSQs total score (week 4: 1.3 vs. 0.1; week 24: 1.8 vs. 0.2; week 48: 1.5 vs. 0.4) and sub-scores for lifestyle/ease (week 4: 0.7 vs. 0.1; week 24: 1.0 vs. 0.1; week 48: 0.8 vs. 0.1) and the general satisfaction/clinical subscale (week 4: 0.6 vs. 0.1; week 24: 0.8 vs. 0.1), except for the general satisfaction/clinical subscale at week 48 [15]. When the total score was analysed by switch phase, participants initially randomised to the CAR arm achieved the same level of treatment satisfaction as those initially randomised to DTG/RPV after a late switch to DTG/RPV at week 52 [15,16].

Phase III data for PWH with stable virological suppression highlights positive experiences with an oral 2DR antiretroviral with a fixed-dose combination of DTG/3TC or DTG/RPV. Moreover, improvements were evident as early as 4 weeks after the switch and were stable through 1 year of the antiretroviral regimen, demonstrating a rapid effect on participants’ perception of treatment that remained constant over time. Although treatment satisfaction data from clinical trials evaluating the efficacy and safety of 2DRsare scarce because PROs and PROMs were not systematically included in study designs until a few years ago, there is unequivocal evidence that simplifying therapy with a two-drug regimen leads to increased treatment satisfaction compared to multi-drug regimens, even from the perspective of PWH.

### 3.2. Treatment Satisfaction in Real-World Studies of Oral Two-Drug Regimens

Real-world data provide valuable insights into treatment satisfaction with an oral 2DR in the management of HIV infection. These studies confirm the benefits of simplified regimens on adherence, tolerability, and long-term treatment satisfaction, ultimately improving patient outcomes and quality of life in real-world clinical practice.

URBAN was a prospective, non-interventional, multi-centre cohort study in ART-naïve and experienced PWH receiving DTG/3TC; overall, 93% were men, the median age was 47 years, and 91% were ART-experienced with a median duration of the previous regimen of 7 years [17]. In pre-treated PWH using DTG/3TC and completing the HIVTSQs at baseline and Year 2, the mean (SD) total HIVTSQs score significantly increased, from 53.5 (±8.4) to 56.7 (±5.1) throughout the study, with a change of 3.2 (±8.1; *p* < 0.001). Sustained treatment satisfaction with DTG/3TC over a 2-year period was also consistent with the fact that the observed regimen discontinuations were not attributed to drug-related reasons [17].

Like URBAN, JUNGLE was a prospective, non-interventional, cohort study in PWH under virological suppression switching to DTG/RPV who were predominantly male and with a median age of 48 years. Among people included in the analysis, the statistically significant increase in mean HIVTSQs score already observed at Year 2 (+4.2; *p* < 0.001) was maintained at Year 3 (+4.7; *p* < 0.001) [18], as illustrated in Table 1.

In JUNGLE, PWH could change treatment whenever they wanted and the treatment persistence at 2 years was 74%, mostly due to participant wishes or study withdrawal [18]. Reasons for switching to DTG/3TC and consequent treatment satisfaction was evaluated in the PAIRED study, a cross-sectional survey supplemented with qualitative interviews of PWH receiving DTG/3TC for at least 3 months. An interim analysis of 100 participants with a median age of 52 years and a mean time since HIV diagnosis of 18 years showed that the most frequent reasons for switching to DTG/3TC were avoiding side effects, minimising long-term effects, and reducing drug accumulation over time. As for the HIVTSQs, 76% had a score of 6/6, indicating that participants were very satisfied with DTG/3TC, and 93% of 4–6/6, meaning that the majority of participants were somewhat satisfied or above [19].

Semi-structured interviews exploring PWH and healthcare professional (HCP) experiences with DTG-based 2DRs were conducted in 39 participants and 8 HCPs in Spain and the US. Considering that most of the participants had been living with HIV for nearly two decades and had considerable exposure with prior multi-drug and multi-pill ARV regimens before switching to DTG + 3TC or DTG + RPV, the main benefits of 2DRs that emerged from the qualitative interviews were fewer concerns about ART toxicity and reduced pill burden [20].

In the PROBI prospective cohort study, which included virologically suppressed participants receiving DTG/3TC in routine care with a previous regimen of at least three drugs, PWH and HCPs had been interviewed to assess the acceptability, management experience, perceptions of treatment, and proposal for treatment change. Common expectations of switching to a 2DR for both PLHIV and HCPs included maintaining regimen efficacy and tolerability, simplifying treatment in daily life, and maintaining or improving quality of life with a single-tablet regimen.

With respect to the newly available treatment strategies, while some PWH felt that oral 2DRs might have fewer benefits than weekly dosing regimens or injectable treatments, others considered oral 2DRs more reassuring because daily dosing ensures adherence and reduces the fear of missing injection appointments with rigid dosing windows [21].

Data obtained using PROs revealed increased treatment satisfaction with oral 2DRs in the real-world setting, showing sustained treatment satisfaction with a simplified regimen over time. PWH continue to report high levels of satisfaction with the convenience, tolerability, and efficacy of their treatment, highlighting the durability of these benefits in real-world settings. Future studies are needed to assess whether treatment satisfaction with these newer regimens will remain stable over longer periods of follow-up.

**Table 1:** Treatment satisfaction of 2-drug regimens in real-world studies.

Author (Year)	Study Participants	Outcomes	Results
Beer et al., 2022 [16]	367 ART- naïve and pre-treated PWH receiving DTG/3TC	Treatment satisfaction assessed by HIVTSQs	Mean (SD) total HIV-TSQs score increased significantly, from 53.5 (±8.4) to 56.7 (±5.1), with a change of +3.2 (8.1; *p* < 0.001) in pre-treated PWH completing questionnaires at baseline and Year 2
Schabaz et al., 2022 [18]	200 PWH on suppressive ART switched to DTG/RPV	Treatment satisfaction assessed by HIVTSQs	Treatment satisfaction score increased at Year 2 [mean change +4.2 (*p* < 0.001)] and at Year 3 [mean change +4.7 (*p* < 0.001)]
Slim et al., 2023 [19]	Interim analysis of 100 PWH switching to DTG/3TC	Treatment satisfaction assessed by HIVTSQs and qualitative interviews (n = 20)	76% were very satisfied with DTG/3TC (score 6/6), 93% somewhat satisfied or above (score of 4–6/6). 85% agreed that DTG/3TC had a better impact on their overall health vs. 3-drug regimens
Petit et al., 2022 [21]	15 PWH on DTG/3TC and 15 HCPs	Treatment satisfaction assessed by qualitative interviews	The interviews highlighted 3 common expectations when switching to dual therapy: maintenance of efficacy, simplification of treatment in daily life, and maintenance of quality of life

3TC: lamivudine; DTG: dolutegravir; HIVTSQ: HIV Treatment Satisfaction Questionnaire; HCP: healthcare professional; PWH: people with HIV; RPV: rilpivirine; SD: standard deviation.

### 3.3. Perceived Bother of HIV Symptoms in Clinical Trials of Oral Two-Drug Regimens

The earlier phase III studies of the 2DR strategy GEMINI-1 and—[22] and TANGO [23] only used PROs to assess general QoL, while the later phase III studies SWORD-1 and -2 [14], SALSA [13], and STAT [24] used PROs to understand the experience of PWH for additional PRO dimensions, including symptom distress. In all three studies, the perceived bother of HIV symptoms has been explored through the HIV Symptom Index [25], a self-administered questionnaire in which PWH are asked to score twenty items, each representing a possible symptom of discomfort in the past four weeks, from 0 to 4 (total score range: 0–80 points).

Participants switching to DTG/RPV in the open-label multi-centre randomised SWORD-1 and -2 studies were mainly male (>75%), about 28% were older than 50 years, and 11% had in their anamnesis an AIDS-defining condition (CDC stage C). All were on their first or second ART regimen and were virologically suppressed at the time of study enrolment. The baseline symptom bother scores were 9.6 (±standard deviation, 10.0) in the DTG + RPV switch group and 11.0 (±11.2) in PWH who continued the CAR. A greater reduction in symptom distress was observed following the switch to DTG + RPV versus continuing CAR at both 4 and 48 weeks, while similar scores were reported at 24 weeks. Also, people originally in the CAR arm reported improvements in symptom bother score after a late switch to DTG + RPV, albeit minor, compared to those first randomised to DTG + RPV [16]. This was in apparent contrast to the proportion of adverse events reported by the SWORD investigators, as high as 77% in the DTG + RPV group and 71% in the CAR group, with more participants taking dolutegravir–rilpivirine (3%) that reported adverse events leading to withdrawal than participants taking CAR (<1%) [14].

The perceived bother of HIV symptoms has also been evaluated in people treated with the 2DR DTG/3TC in SALSA trial. SALSA was an open-label multi-centre randomised study enrolling adults with HIV-1 RNA <50 copies/mL and no previous virologic failure. Participants had a median age of 45 years, and about 75% had more than 500 CD4 + T cells/mmc and were on ART for a median 63 months in the switch group and 71 months in the group continuing CAR. Participants switching to DTG/3TC reported less symptom distress compared to those continuing a 3 or 4DR CAR, as early as four weeks after switching and were stable through one year of treatment [13]. The four-week adjusted between-group (DTG/3TC vs. CAR) difference in the score was more pronounced in people older than 50 years (−1.8, 95%CI-3.6; 0.0) than in younger study participants (−0.8, 95%CI-2.2; 0.7), but in both groups the adjusted mean change from baseline showed an improvement in the score. The most improved items at 48 weeks after switching to DTG/3TC were anxiety (−14%), myalgia/arthralgia (−10%), sadness (−21%), paraesthesia (−8%), sexual problems (−19%), and headache (−9%). A ≥ 25% reduction compared to baseline in symptom distress was observed for 13 items in the DTG/3TC group and for only one item in the CAR group, suggesting how there was room for improvement after switching to 2DR even in people who complained of bothersome symptoms at baseline, also not directly attributed to ART, and who may be candidates for a lower-drug regimen even if they are not completely satisfied with their health status according to their own perception or that of the clinician.

The improvement of self-perceived symptoms reported in SALSA, similar to what was reported for the SWORD studies and discussed above in this paragraph, is in apparent contrast to the reports of adverse events signalled by the investigators who conducted the trial, who reported a similar proportion of participants with at least one adverse event in the DTG/3TC group (73%) and in those continuing the current treatment (70%) [11]. This underscores how perceptions of adverse events may be different in the person taking a given therapy and in his or her caregiver, and how the individual may consider factors that are poorly considered or explored in daily clinical practice but are relevant in daily life. Conversely, some adverse events perceived as such by the physician may have little relevance to the study participant. This conflict of opinions and perceptions underscores, on the one hand, how the use of PROs may be important to understand which treatments are best tolerated, especially in the case of therapies such as ART that need to be continued for a long time; on the other hand, it underscores how the physician’s role remains crucial in caring about even those aspects that are less intuitive to the person with HIV but that nevertheless require attention and reporting. Some of the perceived improved symptoms captured by PRO questionnaires may not depend on an actual pathogenetic mechanism underlying the resolution of the problem with one therapy rather than another, but on people’s perspectives on the potential benefits of DTG-based 2DRs and overall satisfaction with the switch to a lower-drug regimen and positive reinforcement of therapeutic success over time. Assessments of the perceived bother of HIV symptoms in double-blind trials are not currently available, so the possibility of an effect of satisfaction with the 2DR strategy rather than a significant change in adverse events attributable to therapy remains for the items evaluated in the score.

**Figure 1:**
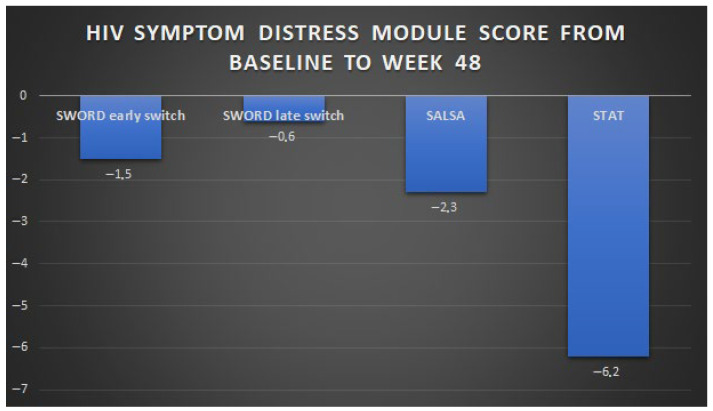
Changes in HIV symptom distress module score from baseline to week 48 in phase III trials of DTG-based 2DRs: SWORD-1 and -2, SALSA, and STAT. Note: For the group of participants enrolled in the SWORD study who were randomised to late switch to dolutegravir + rilpivirine, HIV symptom distress module scores were collected 52 weeks after the switch. HIV symptom distress module scores are instead reported at week 48 for SWORD, SALSA, and STAT participants.

The perceived bother of HIV symptoms has also been evaluated in treatment-naïve PWH initiating DTG/3TC in the multi-centre, single-arm STAT study, in a US test-and-treat setting [24]. Participants in STAT were ART-naïve adults (median age 30 years) with a newly confirmed HIV-1 diagnosis, no prior history of hepatic or renal impairment, and no known or suspected HBV co-infection; a total of 37% had less than 200 CD4 + T cells/mmc. Of the 20 symptoms evaluated, 19 were reported as bothersome at baseline by ≥5% of participants, of which the most frequently reported were difficulties in sleeping, fatigue/loss of energy, feeling sad, down, or depressed, feeling nervous or anxious, and having fever, chills, or sweats. All these symptoms improved by week 4, and overall, the baseline mean score of 13.8 (±standard deviation, 14.7) decreased to 8.5 (±11.3) at week 24 and to 7.7 (±11.1) at week 48 under treatment with DTG/3TC [24]. ART-naïve PWH had a greater improvement in symptom distress compared to ART-experienced participants enrolled in SALSA and SWORD-1 and -2 trials, probably due to the additive beneficial effect of viral clearance and of reduction in concerns related to the newly diagnosed infection, in the course of an effective treatment. The changes in self-perceived symptoms described in 2DR phase III studies are summarised in Figure 1.

### 3.4. Perceived Bother of HIV Symptoms in Real World Studies of Oral Two-Drug Regimens

Despite PROs from interventional clinical trials indicating positive people experiences with DTG-based oral 2DRs, data from routine clinical care are still scarce and will be equally important to confirm these findings. Two main studies have included symptom distress assessment among their outcomes, namely the URBAN and JUNGLE observational studies [17,18]. The final data of these studies are still unpublished at the date of writing; however, preliminary results have been presented in *HIV Drug Therapy Glasgow 2022* [17]. In the URBAN study, the total HIV Symptom Index scores did not change significantly in 144 ART-experienced participants, from 14.0 (±standard deviation, 11.5) to 12.5 (±11.0) and 13.0 (±11.9) at one- and two-year follow-up; and in 11 treatment-naïve participants, from 12.1 (±standard deviation, 14.1) to 10.1 (±16.7) and 12.2 (±12.6) at one- and two-year follow up [17]. In the JUNGLE study, symptom distress module scores did not change significantly from baseline at either the Year 2 or Year 3 evaluations, with mean change of -1.6 (*p* = 0.085) and −0.9 (*p* = 0.163), respectively [18]. The main results of symptom distress changes in observational studies are summarised in Table 2. Future research in non-interventional clinical studies will be critical to understand the perceptions of PWH treated with 2DR and gain insights to build an increasingly person-centred approach to ART, aimed at the goal of long-term success not only from a virological perspective, but also in terms of the quality of life of the person on therapy.

### 3.5. Cabotegravir and Rilpivirine Treatment Satisfaction and Bother Symptoms in Clinical Trials

In a qualitative study conducted among participants enrolled in the phase II study of CAB + RPV (LATTE-2) in the United States and Spain, 39 in-depth interviews were conducted to explore participant and provider attitudes and experiences with injectable long-acting (LA) versus oral ART [26]. Twenty-seven trial participants, from the LA 4- or 8-week arms, and twelve providers were recruited from LATTE-2 sites in the US and Spain. Semi-structured interviews were conducted with a flexible guide of open-ended questions to explore participant views and experiences related to LA treatment. Injection experiences, perceived advantages and disadvantages of daily oral and LA injectable ART, perceived appropriate candidates for LA ART, and future service delivery preferences were the main topics of discussion. Of note, females and non-MSM were underrepresented in the sample. Participants in the interviews described the convenience of LA treatment when compared to the daily pills and emotional benefits, for example, minimised potential for HIV disclosure and eliminating the daily reminder of living with HIV as potential advantages of LA treatment when compared to oral daily regimens. Providers recognised benefits but raised concerns regarding LA candidates, in particular, the requirement of adherence to clinic visits for injections and about the changes in actual clinical management. Overall, LA treatment was generally identified as preferable to daily oral ART among persons with HIV interviewed [26]. The phase III ATLAS [27] and FLAIR [28] studies demonstrated that maintenance with monthly LA intramuscular CAB + RPV is non-inferior to continuing the current antiretroviral oral therapy. In the same study, apart from the primary efficacy endpoint, several other exploratory endpoints related to health status, treatment satisfaction, acceptance and preference, and the tolerability and acceptability of injections were investigated. The choice of instruments to assess such outcomes was based on the above-mentioned qualitative interviews conducted during the LATTE study [26]. Treatment satisfaction was assessed by means of a new version of the 10-item HIV Treatment Satisfaction Questionnaire (HIVTSQ) [10,29] including two additional questions: (1) How easy or difficult have you been finding your treatment to be recently? (2) How satisfied are you with the amount of discomfort or pain involved with your present form of treatment? The HIVTSQs (status version) asked patients to rank their response on a 6-point Likert scale, from 6 (very satisfied) to 0 (very dissatisfied), and the questionnaire was administered in both treatment arms at 4, 24, and 44. The HIVTSQc (change version) was administered only at week 48 to both treatment groups in FLAIR and to the LA group only in ATLAS. The ACCEPT questionnaire was administered at baseline, week 8, week 24, and week 48 to assess the treatment acceptability. The perception of pain and injection site reaction was assessed by means of the PIN questionnaire at weeks 5, 41, and 48. The general health status and degree of mental health distress was assessed by means of SF-12 at baseline and at weeks 24 and 48 in both studies. A single preference question was used in both studies at week 48 to assess the preference of LA when compared to oral treatment. In the end, only in the ATLAS study was a single question regarding the reason of switch administered at day 1 in those randomised and at week 52 in those who chose to switch to LA after the 48 weeks’ completion [27,28]. ATLAS and FLAIR were pooled and the final intention-to-treat-exposed (ITT-E) population of all randomised participants who received at least one dose of study medication consisted of 1182 individuals: 591 in each treatment group [LA or current antiretroviral regimen (CAR) [30]. The characteristics of the study participants in the two studies mainly differ due to a higher percentage of people aged above 50 in the CAR group in ATLAS and because participants enrolled in ATLAS had been on previous cART for a median of 4 years (range 1–21) [27], while participants in FLAIR had only a previous induction therapy before LA [28]. The mean HIVTSQs total score values at maintenance baseline were high in both arms in ATLAS and FLAIR, with higher mean scores in FLAIR. Treatment satisfaction significantly improved in the pooled analysis at weeks 24 and 44 in the LA arm when compared to CAR. This difference observed between LA and CAR in the pooled data was mainly driven by ATLAS results, with the LA arm showing a 5.4 (95% CI 4.2–6.6) to 5.7 (95% CI 4.4–7.0) point treatment difference in the adjusted mean change from maintenance baseline in HIVTSQs total score vs. CAR arm. Of note, the difference observed was deemed clinically significant due to exceeding one half of the standard deviation at baseline [30]. The magnitude of the improvement in treatment satisfaction was lower in the FLAIR study with a significant difference (*p* < 0.001) in mean HIVTSQc total score observed for the LA arm [adjusted mean 29.6 (standard error (SE) 0.49)] vs. CAR [ adjusted mean 25.2 (SE 0.48)] but no difference in HIVTSQs at week 48 (*p* = 0.217).

Regarding the treatment acceptance at baseline in both FLAIR and ATLAS, high mean values were observed. For HIVTSQs, a significant improvement was observed in the LA arm when compared to CAR in the pooled analysis [adjusted pooled difference 5 (95% CI 2.4–7.5) at week 24 and 6.8 (95% CI 4.2–9.4) at week 48]; again, this improvement was mainly driven by the ATLAS study results [adjusted pooled difference 6.9 (95% CI 3.3–10.4) at week 24 and 10.7 (95% CI 7.1–14.4) at week 48] with FLAIR point estimates not showing significant differences in acceptance at week 24 (*p* = 0.154) and 48 (*p* = 0.236) [27,28].

The acceptability of injection site reaction was high in both studies at week 5 and in addition, in the analysis of PIN, a statistically significant mean improvement from week 5 in the acceptance domain was observed in pooled data at weeks 41 [mean 1.67 (standard deviation (SD) 0.86)] and 48 [1.62 (SD 0.81)], suggesting an improved acceptability of injection site reactions over time (*p* < 0.001) [27,28].

In both FLAIR and ATLAS, no significant changes in SF12 were observed from baseline to week 24 and 48, although the score ranked generally high from the baseline when compared to the US national average [27,28,31].

Regarding the preference of LA treatment, only 2% (7/308) in ATLAS and 1% (2/283) in FLAIR preferred daily oral treatment at week 48—a finding which was supported by a post hoc analysis in which almost all participants at week 48 preferred the LA treatment when compared to CAR (97% in ATLAS and 99% in FLAIR) [27,28].

The most frequent answers regarding treatment preferences at baseline were related to interest in research (82%) and suggestions by in-charge physicians (25%) in ATLAS. When the choice to switch to LA was offered at week 52, the convenience (53%) and discretion (32%) provided by LA emerged as the main reasons for switching [27].

In the ATLAS-2M study, the non-inferiority of CAB + RPV LA administered every 8 weeks (Q8W) versus every 4 weeks (Q4W) has been demonstrated in the maintenance of virologic suppression [32]. In the same study, the authors’ participants’ experience with the two dosing regimens of CAB + RPV LA (Q4W and Q8W) was compared using PRO data from preplanned and post hoc analyses [33]. The intention-to-treat-exposed population consisted of 1045 participants (522 in Q8W and 523 in Q4W). Overall, 37% (391/1045) of participants entered ATLAS-2M with prior CAB + RPV experience in ATLAS, most of whom (65%, 253/391) had more than 48 weeks of prior exposure to CAB + RPV LA. No statistically significant difference in adjusted mean change from week 8 to weeks 24 and 48 in the acceptability of injection site reactions was observed between the LA arms (*p* = 0.768 and *p* = 0.391 at week 24 and 48, respectively). For participants previously enrolled in ATLAS, the pain perception of the injection site remained substantially stable. On the contrary, a significant improvement (*p* < 0.001) from week 8 to weeks 24 and 48 in the acceptability of injection site reactions were observed for participants with no prior exposure to CAB + RPV LA across both LA arms [33]. No significant differences were observed between the two LA arms in change from baseline in treatment acceptance at weeks 24 and 48 (*p* = 0.379 and *p* = 0.525, respectively). For those not previously exposed to CAB/RPV LA at week 24 and week 48, marked improvements from baseline in treatment acceptance were observed across both long-acting arms [adjusted mean change from baseline Q8W: week 24, 5.8 (95%CI 3.2–8.5); week 48, 6.8 (95%CI 4.3–9.3); Q4W: week 24, 4.2 (95%CI 1.5–6.8); week 48, 5.7 (95%CI 3.2–8.1)] [33]. For participants without prior CAB/RPV exposure, at both week 24 and week 48, HIVTSQs total scores were markedly improved from baseline (adjusted mean change from baseline Q8W: week 24, 5.07 (95%CI 4.36–5.78); week 48, 4.86 (95%CI 4.02–5.69); Q4W: week 24, 4.00 (95%CI 3.29–4.70); week 48, 3.12 (95% CI 2.29–3.95)], with significant differences in the adjusted mean change from baseline for Q8W dosing at both week 24 (*p* = 0.036) and week 48 (*p* = 0.004) when compared to Q4W dosing. On the contrary, no significant differences (*p* > 0.05) in HIVTSQs were found between the long-acting groups at any time point. At week 48, the change in satisfaction relative to the prior treatment was greater for Q8W when compared to Q4W, irrespective of being previously exposed to CAB + RPV LA [adjusted difference between Q8W and Q4W: participants without prior CAB + RPV LA exposure, 1.9 (95% 0.5–3.2), *p* = 0.008; participants with prior exposure, 2.9 (95% 1.0–4.8), *p* = 0.004] [33].

Almost all participants previously exposed to CAB/RPV LA preferred the Q8W (94%) when compared to Q4W (3%) or daily oral dosing (2%). The responses were similar for those unexposed at baseline to CAB + RPV LA, with 98% and 94% of individuals preferring CAB + RPV LA Q8W and Q4W at 48 weeks, respectively [33].

Interest in new treatment was the main reason for switching for those unexposed to CAB + RPV LA at baseline (85%), whereas convenience was reported by 87% of individuals already on CAV + RPV LA [33].

In the SOLAR study (a randomised, open-label, phase IIIb, non-inferiority trial), the efficacy, safety, and tolerability of switching to LA CAB + RPV versus continuing fixed-dose bictegravir, emtricitabine, and tenofovir alafenamide in virologically suppressed adults with HIV has been evaluated [34]. Six hundred and eighty-seven participants were randomly assigned to switch treatment or continue existing treatment. At month 11–12, LA CAB/RPV showed non-inferior efficacy versus bictegravir, emtricitabine, and tenofovir alafenamide [HIV-1 RNA ≥ 50 copies per mL, five (1%) of 447 vs. one (<1%) of 223]. The investigated patient-reported outcomes included change in treatment satisfaction (HIVTSQs) at month 11 (LA without OLI) or month 12 (LA with OLI and bictegravir, emtricitabine, and tenofovir alafenamide) and participant treatment preference evaluated by means of a preference questionnaire at month 11 (LA without OLI) or month 12 (LA with OLI and bictegravir, emtricitabine, and tenofovir alafenamide) or withdrawal. Participants’ emotional wellbeing and adherence considerations were evaluated at baseline [34].

At baseline, 315 (47%) of 670 participants across the LA and bictegravir, emtricitabine, and tenofovir alafenamide groups reported having a fear of disclosure reported as always/often, adherence anxiety, or a daily reminder of HIV status. Treatment satisfaction was greater among participants in the LA arm compared with those in the bictegravir, emtricitabine, and tenofovir alafenamide arm, with a significant improvement in satisfaction observed through to month 11–12 in the LA arm. The mean adjusted HIVTSQs scores improved significantly for LA versus bictegravir, emtricitabine, and tenofovir alafenamide participants from baseline to months 5–6 [LA, +3.86 (95% CI 3.14–4.57)]; bictegravir, emtricitabine, and tenofovir alafenamide, −0.4 (−1.41 to 0.61); *p* < 0.001] and months 11–12 [LA, +3.36 (2.59 to 4.13); bictegravir, emtricitabine, and tenofovir alafenamide, −1.59 (−2.71 to −0.47); *p* < 0.001] [34].

Most participants in the LA arms (90%, 382/425) reported a preference for CAB + RPV LA instead of oral treatment at month 11–12 with the remaining 22 (5%) of 425 having no preference and 21 (5%) of 425 reporting a preference for daily oral therapy [34].

Among the reasons for preferring LA therapy instead of oral treatment, the most frequent were not having to worry about remembering to take HIV medicine (85%), convenience (83%), not having to carry HIV medication (74%), not having to think about HIV status every day (61%), and not having to worry about others seeing or finding HIV pills (59%) [34]. A summary of PROs in RCTs investigating CAB/RPV is reported in Table 3.

### 3.6. Comparative Overview of Oral vs. Injectable 2DRs Based on PROs

Table 4 presents an integrative comparison of patient-reported outcomes (PROs) across oral and injectable dual antiretroviral regimens (2DRs). Oral regimens are generally associated with concerns related to daily pill burden, gastrointestinal tolerability, and the risk of missed doses, but offer advantages in autonomy, convenience, and flexibility. In contrast, injectable regimens reduce the need for daily adherence and may be preferred by patients with adherence challenges, although they introduce concerns around injection discomfort, clinic visit frequency, and injection-site reactions. Satisfaction drivers differ across modalities, reflecting both lifestyle and psychosocial factors, while patient populations demonstrate distinct preferences: those valuing independence often favour oral therapy, whereas individuals facing adherence barriers may benefit more from injectables. Practical constraints, including visit scheduling, disclosure considerations, and resource availability, further shape the patient experience. By synthesising these domains, the table provides a clear framework for understanding how regimen type influences PROs and can guide clinicians in tailoring treatment decisions to individual patient needs.

### 3.7. Implementation of the Use of PROs in Clinical Practice: Obstacles and Possible Solutions

Both EMA and DHHS guidelines have recommended the use of PROs for the evaluation of new treatments and optimisation of antiretroviral therapy, given their relationship with therapeutic efficacy. PROs can play a decisive role in the choice of ART regimens since, given equal levels of efficacy and safety, a better assessment of the level of satisfaction, compliance, and comfort with treatment can help to determine the most appropriate regimen. On the other hand, PROs help to explain certain discrepancies between the results of clinical trials and those obtained in real life [35]. Therefore, incorporation of PROs into routine clinical practice places PWH at the centre of therapeutic decisions, as it not only allows the identification of patients’ problems and concerns but also facilitates more efficient and personalised healthcare. The benefits of the implementation of PROs will also result in a better allocation and efficiency of healthcare resources, which is essential in today’s healthcare systems. Moreover, there is a significant perception gap regarding the use of PRO between PWH and their physicians. PWH positively value them and demand the use of simple tools written in a language appropriate to their situation. On the contrary, health professionals find it difficult to include the use of questionnaires collecting PROs in clinical practice [4]. Among the reasons for not implementing PROs, the lack of time in the consultation room stands out together with the lack of other professional figures to be in charge of their completion. In addition, it has been observed that health professionals underrate information on the usefulness and benefits of PROs specific to PWH in multiple health domains, for example, quality of life, adherence, social factors, or satisfaction with care. The above-mentioned barriers are solvable and a rapprochement between the attitudes of healthcare professionals and PWH should be encouraged to achieve a consensus that can improve the quality of life of these people.

All the questionnaires used to assess PROs need to be validated and adapted to the local context in order to be useful. A differentiated use of PROs between newly diagnosed and follow-up PWH may help to pursue an individual-centred care approach, as proposed by the PROMETEO project. In newly diagnosed cases the use of general multidimensional PROs is recommended, preferably in paper format to facilitate a more personalised conversation, while in PLHIV attending follow-up visits, PROs for the assessment of specific areas are preferable and electronic format is favoured to study their evolution [4].

PRO questionnaires should also be brief, clear, and concise; they should include some open-ended questions because sometimes PWH do not feel identified with some of the answers and should be self-administered, so that answers are not influenced by the presence of the healthcare personnel. On some occasions, a training for completion is required because of the complexity of some questions. The presence of nursing staff, educators, patient associations, or other health centre personnel with specific training could help in the completion of the PROs, particularly with older people, patients with cognitive impairment, or migrants who may have barriers due to language difficulties. Electronic collection of PROs is becoming increasingly popular as it reduces completion time and the likelihood of errors, allowing the results to be used on an individual basis or in series [36].

In a review by Campbell, the use of tablet-based PRO assessments was an important factor in the high acceptability of PROs [37]. Patients generally prefer tablet-based over paper-based administration and the ability to integrate skip patterns into the design of the assessment (e.g., not asking nonsmokers about smoking patterns) ensures a manageable time burden on patients and decreases impact on patient flow. Electronic questionnaires can rapidly generate summary results, record and track changes in health status, encouraging engagement and self-care between visits [38]. The availability of longitudinal health data, including changes not only on health-related parameters but also PROs, may even encourage those who dislike questionnaire collecting PROs. Some devices also provide a graphical overview so that data may also be relatively easy to interpret. This implementation could also improve the response rate of PROs which are usually lower in clinical routine compared to a protocolled research setting.

The completion of PROs collected before the appointment could be useful for prioritising discussion topics with their physicians, helping to initiate discussion on sensitive issues and improving comprehensiveness and satisfaction with care. In addition, PROs could identify PWH with severe conditions such as depression and may reveal suicidal ideation, worthy of timely intervention. In electronic form, these symptoms could be promptly flagged or highlighted and reported by an alarm system so that an intervention can be immediately taken. With a digital format, it is also possible to include an audio component for PWH with poor vision or low literacy. A results summary can be generated in the provider’s desired language regardless of the language in which the assessment was administered to the patient [39]. Despite the advantages of electronic systems for completing questionnaires through cell phone applications, web pages, e-mails, etc., their use in clinical practice is not yet well established [40]. One of the reasons may be the lack of electronic devices among some PWH, especially the elderly who are at risk of social exclusion. However, the possibility of completing PROs on electronic devices does not imply the abandonment of the paper format, as there are still PWH with a clear preference for the latter. A cost analysis of PRO implementation in clinical practice has estimated a low initial cost for the purchase of electronic devices (tablet computers/iPads) and printing costs where the assessment report was delivered on paper, but larger costs are required for human resources, including time for setup, training, monitoring, and reviewing. However, once the PRO program is established, each PRO assessment including patient engagement, monitoring, tablet preparation, and analysis of results will require progressively less time and resources [39].

Costs analyses must also consider that some validated questionnaires are protected by copyright and are available under payment. The use of patient portals or URLs has the potential to open access to PROs to a wider range of users, including those with restricted access.

## 4. Discussion

Different international guidelines recommend the use of PROs for the evaluation of new treatments and optimisation of antiretroviral therapy, but they do not define the specific kind of questionnaire, the timing, and the methods of administering the same questionnaires to different persons. According to our narrative review, the 2DR, both oral and injectable, seems to be linked to a higher treatment satisfaction according to the PROs collected with validated instruments. Nevertheless, according with the results of studies including PROs as outcomes, the results of the studies are difficult to interpret. The clinical trials, which provided most data regarding PROs, reflect this information gap, because they are not homogeneous for the choice of specific questionnaire. Some of these used qualitative interviews, others used HIVTSQc, and others used semi-structured interviews conducted with a flexible guide of open-ended questions. Furthermore, the methods of administration of the questionnaire are quite different: the PROs could be paper or online, they could be completed or not in the hospital during the time point, they could be written in one or more different languages for each PWH, and they could be self-administered or completed with the help of dedicated nursing and/or medical staff. This different information could affect the results. PRO questionnaires should also be brief, clear, concise, and easy to complete. They should include some open-ended questions to help PWH to express their sensations and emotions. It should be self-administered, so that answers are not influenced by the presence of the healthcare personnel. On the other hand, the presence of nursing staff, educators, patient associations, or other health centre personnel with specific training could help in the completion of the PROs, particularly with older people, patients with cognitive impairment, or migrants who may have barriers due to language difficulties. Beyond the clinical utility of PROs, psychological and behavioural aspects also emerge as relevant biological determinants of treatment perception among PWH. Several studies included in this review indirectly suggest that simplified dual regimens may modulate anxiety and treatment-related fears. In qualitative interviews and PRO analyses from the LATTE-2 and SOLAR trials, participants reported that long-acting injectable therapy reduced daily reminders of HIV infection and alleviated anxiety related to adherence and disclosure, while a minority expressed concerns about injection scheduling or potential viral rebound. Conversely, in switch studies of oral 2DRs such as SWORD and SALSA, participants frequently cited improved confidence in therapy durability and fewer worries about long-term toxicity, although some residual apprehension about resistance or viral breakthrough persisted, especially among those with previous treatment failures. Gender-specific analyses remain limited, but the available data suggest similar overall treatment satisfaction between women and men, with some reports indicating slightly higher convenience and adherence scores among women—possibly reflecting greater concern for treatment flexibility and privacy. Finally, differences in PROs were observed between ART-naïve individuals initiating a 2DR and virologically suppressed participants switching from 3DR regimens: in the STAT study, treatment-naïve patients experienced more pronounced improvements in symptom distress and psychological wellbeing, likely due to relief associated with viral suppression and reduced disease perception, while switch populations mainly reported stable or modestly improved satisfaction levels. Taken together, these findings highlight that the psychological dimension of simplification—reduced pill fatigue, fear of stigma, and improved sense of control—represents a key mediator of patient-reported outcomes and should be systematically integrated into future biological and behavioural research frameworks on antiretroviral treatment optimisation.

This narrative review has several inherent limitations. Unlike systematic reviews, our search strategy was not fully standardised, and formal appraisal of study quality was not performed, which limits the ability to evaluate the robustness of individual trials or PRO assessments. The included studies varied in design, population, and PRO instruments, and these heterogeneities were addressed qualitatively rather than quantitatively, potentially affecting the generalisability of our conclusions. Finally, because narrative reviews do not follow strict systematic protocols, the results provide a broad overview of PRO assessment in dual antiretroviral regimens (both oral and injectable) but should be interpreted with caution when informing clinical decision-making or guideline development.

Despite the benefits of PRO implementation within clinical care and guidelines recommendations, the effective integration into care settings is still largely restricted to clinical trials and an information gap still exists—particularly in a non-experimental context. Looking ahead, several critical areas remain to be addressed for PROs to achieve full clinical and policy impact in HIV care. First, there is still no consensus on the most appropriate instruments for specific settings—such as routine follow-up visits, clinical trials, or implementation studies—nor on the level of granularity required to balance psychometric validity with feasibility in busy clinics. Comparative research identifying context-adapted, time-efficient PRO tools is therefore needed. Second, harmonisation of PRO endpoints across HIV clinical trials is a priority to improve comparability, meta-analytic synthesis, and regulatory acceptance of patient-centred outcomes. Third, PROs have the potential to influence both clinical guidelines and reimbursement frameworks, as they provide measurable data on patient wellbeing, satisfaction, and adherence—domains increasingly valued by health authorities and payers. Integration of standardised PRO metrics into regulatory submissions could support the evaluation of treatment value beyond viral suppression. Finally, from a real-world perspective, sustainable implementation will require digital solutions, dedicated personnel, and workflow redesign to minimise burden on clinicians while ensuring meaningful use of patient feedback. Addressing these challenges through coordinated academic–industry–policy collaboration will be essential to move PROs from research tools to actionable components of quality HIV care. In conclusion, our review shows that there are multiple positive signals in the literature on the use of PROs in clinical practice in the setting of the optimisation of ART to a 2DR in virologically suppressed PWH. Although trials show us that their use can reveal various useful information about the treatment and the wishes of people in therapy, there remain many gaps that future research must try to fill. It remains to be established which questionnaires to systematically use to evaluate PROs, particularly in relation to individual characteristics; stakeholders will have to worry about establishing dedicated pathways in HIV clinics for the evaluation of PROs, dedicating resources, spaces, and dedicated personnel who have received adequate training.

## Figures and Tables

**Table 2:** Real-world studies of oral 2-drug-regimens: key PRO messages about perceived bother of HIV symptoms in pre-treated people with HIV.

	Pre-Treated PWH Receiving DTG/3TC (URBAN) (Beer et al., 2022) [17]	Virologically Suppressed PWH Receiving DTG/RPV (JUNGLE) (Schabaz et al., 2022) [18]
Symptom distress	Participants’ symptom distress remained relatively stable over 2 years of DTG/3TC use	
Mean change from BL to Year 2 (SD)	Mean BL score (SD): 14.0 (11.5) Mean change from BL to Year 2 (SD): −1.0 (11.4) *p* value for change from baseline *p* = 0.26	Mean BL score (SD): NR Mean change from BL to Year 2 (SD): −1.6 (NR) *p* value for change from baseline *p* = 0.085

3TC: lamivudine; BL: baseline; DTG: dolutegravir; PWH: people with HIV; NR: not reported; RPV: rilpivirine; SD: standard deviation.

**Table 3:** Summary of PROs in RCTs investigating CAB/RPV treatment in PWH.

Author, Year	Phase	Intervention and Comparison	N Participants	Type of End Point	Type of PRO Instrument	Outcome	Main Findings
Kerrigan et al., 2018 [26]	Phase IIB	(1) LA injections every 4 weeks, (2) LA injections every 8 weeks, or (3) continue on the daily oral regimen	27/309	Exploratory	-Open-ended questionnaire on self-reported experience	-Patient preferences at 4 or 8 week (cross-sectional)	Participants emphasised greater convenience versus daily pills and reported emotional/psychosocial benefits, notably the reduced risk of inadvertent HIV disclosure and removal of the daily “reminder” of living with HIV (a key driver of satisfaction and perceived wellbeing). Providers acknowledged potential benefits but highlighted implementation concerns that can affect patient experience, especially the need for reliable attendance at clinic visits for injections and the implications for routine clinical management/workflows.
Swindells et al., 2020 [27]	III open label	PI/b or INSTI or NNRTI + 2NRTIs vs. monthly CAB + RPV	618 (1:1)	Secondary	-HIVTSQs/c -Single-item question for LA preference	-Satisfaction with patients’ CAR vs. LA at BL and week 24 and 44 -LA preference vs. CAR at week 48	Participants receiving CAB + RPV LA reported significant improvements in treatment satisfaction and greater convenience compared with those remaining on oral therapy. PRO scores indicated less treatment-related anxiety, reduced daily reminder of HIV, and a strong preference for injectable therapy (over 90% preferred the long-acting regimen). Quality-of-life (SF-12) scores remained stable or improved, with no signal of worsening mental or physical health domains.
Orkin et al., 2021 [28]	III open label	ABC/3TC/DTG vs. monthly CAB + RPV	566 (1:1)	Secondary and exploratory	-HIVTSQs/c -Single-item question for LA preference	-Change version HIVTSQc at week 48 -LA preference vs. CAR at week 48	Participants consistently reported reduced pill fatigue, less anxiety about missed doses or HIV disclosure, and a greater sense of freedom and control over their treatment. More than 90% of participants expressed a preference for the injectable regimen, citing improved privacy and reduced daily reminder of HIV infection.
Murray et al., 2020 [30]	Phase III open-label pooled analysis	-PI-, NNRTI-, INSTI- +2 NRTI vs. monthly CAB/RPV -ABC/3TC/DTG vs. monthly CAB/RPV	618 (1:1) 631 (1:1)	Secondary and exploratory endpoint	-HIVTSQs/c -ACCEPT -PIN -SF-12 -Treatment preference -Reason for switch -HAT-QoL -Numeric rating scale	-Treatment satisfaction BL, weeks 4, 24, 44 and 48 -Treatment acceptance BL, weeks 8, 24 and 48 -Pain perception, weeks 5, 41 and 48 -General health Bl, weeks 24 and 48 -Preference at week 48 -Reasons of switch at week 52 -Overall function at BL and weeks 24 and 48 -Intensity of pain at weeks 4, 5, 40, and 41	Participants switching to CAB + RPV LA reported significant improvements in treatment satisfaction and convenience compared with those remaining on daily oral therapy. Across 48 weeks, scores on the HIV Treatment Satisfaction Questionnaire (HIVTSQs and HIVTSQc) increased markedly, reflecting reduced treatment burden and greater perceived flexibility. Participants also reported less anxiety about adherence and disclosure, as the injectable regimen eliminated daily reminders of HIV. Measures of health-related quality of life (SF-12) and injection site tolerability remained stable or improved, with >90% of participants preferring the long-acting regimen over oral therapy at week 48. Overall, PRO data demonstrated high satisfaction, strong preference for long-acting dosing, and minimal negative impact on QoL across diverse demographic subgroups.
Chounta et al., 2020 [33]	Phase IIIB	CAB/RPV every 4 vs. 8 weeks	1045	Secondary and exploratory endpoint	-HIVTSQs/c -ACCEPTANCE -PIN -Treatment preference -HAT-QoL -Reason for switch	-Treatment satisfaction at BL and weeks 24 and 48 -Treatment acceptance at BL and weeks 24 and 48 -Pain perception at weeks 8, 24, and 48 -Preference at week 48 -Reasons for switch BL -Overall function at BL and weeks 24 and 48	Comparable or greater satisfaction with q8-week dosing; minimal injection-related anxiety.
Ramgopal et al., 2023 [34]	Phase IIIB	Switch to Cab/RPV vs. BIC/TAF/FTC	837	Exploratory endpoint	-HIVSTQs -Treatment preference -Emotional wellbeing and adherence	-Treatment satisfaction at months 11 or 12 -Preference at month 11 or 12 -Wellbeing and adherence BL	Significant improvement in satisfaction scores; reduced daily pill burden perceived as major advantage.

**Table 4:** Framework of patient-reported outcomes in oral vs. injectable 2DRs.

Domain	Oral 2DRs	Injectable 2DRs	Comments/Implications
Patient concerns	Pill burden, daily adherence, gastrointestinal tolerability	Injection-related discomfort, visit frequency, injection-site reactions	Oral may be preferred for self-management; injectable may be preferred by patients struggling with daily adherence
Drivers of satisfaction	Convenience, autonomy, flexibility in timing, minimal clinical visits	Reduced daily medication reminders, discreet administration, perceived innovation	Satisfaction influenced by lifestyle, adherence patterns, and social context
Population preferences	Patients valuing independence, with stable routines	Patients with adherence challenges or cognitive/behavioural difficulties	Tailoring modality choice to person characteristics may improve PROs and adherence
Practical constraints	Requires daily self-management, disclosure risk in shared households	Clinic visit scheduling, potential for missed appointments, injection anxiety	Implementation considerations critical for clinics with resource constraints
Adherence and anxiety	Risk of missed doses, pill fatigue	Anxiety around injections or visit scheduling, but fewer daily reminders	PROs reflect both regimen burden and psychological factors

## Data Availability

All data generated are available herein.
